# A Simple Remedy

**Published:** 1876-07

**Authors:** 


### *A SIMPLE REMEDY.*

We are treating a case of what is popularly known as "salt rheum." It seemed to resist all the usual remedies for weeks. We finally remembered that when a boy we were frequently *wounded* in our battles with the bumble bees during hay-making. A little mud applied to the spot would allay the pain immediately. So we procured some common clay, softened it with water, and applied it as a poultice. Its effect was magical. The itching, which was at times almost unendurable, was at once allayed, and now, having used it constantly for five or six days, the salt rheum seems to be cured. There is no deodorizer more potent than fresh earth ; nothing that will more thoroughly cleanse an ulcer than a clay-plaster, and its healing qualities are probably equal to its disinfecting properties. It is a remedy that costs nothing, and is always at hand.

